# Mr. Harrold's Case of Paralysis

**Published:** 1812-12

**Authors:** E. Harrold

**Affiliations:** Cheshunt, Herts


*To the Editors of the Medical and Physical Journal.*

GENTLEMEN,

HAVE the goodness to insert in your Journal, if you think it deserves a place there, the following case of Paralysis, which seems interesting on account of the evident connection which subsisted between the diseased state of the nerves and the condition of the stomach.

On subjects of this kind, there has been of late some diversity of opinion amongst medical men; and the record of a few important facts, may throw a clearer light upon the kindred diseases—palsy and apoplexy—which may also lead to a more decided and appropriate practice.

Dr. Cheyne's book upon the latter disease, judging of its contents by what I have at present seen of it, as quoted in your Journal, establishes, upon the most solid grounds, the existence of a very disproportionate quantity of blood in the vessels, &c. of the brain. Some have been hardy enough to argue in opposition to these *facts*, that the cranium can never contain more or less than a given quantity of blood, not considering how much elastic, and of course compressible, vapour, pervades the ventricles, and is interposed between the membranes and convolutions of the brain.

The history of this case, so far as a solitary case goes, serves to prove that palsy and apoplexy, though related, are, in some important respects, very different diseases; and sometimes require a very different mode of treatment.

Mr. Edward Glover, carpenter, æt. 52, (a man very highly respected by people of all classes in this neighbourhood, for his regular habits, punctuality in business, &c. &c.) After having for some days had obscure feelings of indisposition, with a sallowness of complexion, was suddenly seized, on the 3d of March last, with excessive giddiness, as he sat at his desk, and with hemiplegia of the left side. He was with some difficulty carried up stairs. I visited him directly, but found, on my arrival, that a nearer medical neighbour had given him some eau-de-luce, knowing that his habit was gouty. I found his pulse exceedingly feeble, irregular, and indistinct; and he complained of an icy coldness of the affected side. In order to assist in rousing him from this torpid state, I gave him, what was immediately at hand, a wine glass of hot strong brandy and water; this, fortunately, in a very few minutes operated as an emetic; and, immediately after his stomach was cleared, he exclaimed, "Thank God! I have got the use of my arm and leg again!" His memory was a good deal impaired, but this was not a new circumstance. In the earlier part of his life he had suffered greatly greatly from rheumatism, and afterwards from a sort of irregular gout, sometimes affecting his stomach rather severely. He had at this time not sustained an attack of that sort for several years.

A blister was applied between his shoulders, and an active purgative immediately administered; the affected parts bathed with camphor liniment and ammonia; and, internally, aromatic confection with gr. vj of the carbonate of ammonia 6tis horis. On the 5th, he had one grain of the submuriate of mercury at night, and the purgative repeated on the morning of the 6th; the other remedies continued. On the 7th, as he complained of some oppression at the stomach, he had a cummin-plaster applied across it, and infus. gentian. with the ammonia, internally. On the 9th, the purgative was ordered to be repeated occasionally; the calomel, having affected his bowels rather too powerfully, was not repeated.

On the 11th, he had another attack, not quite so severe as the first, and depriving him of the use of his *right* arm. On this occasion I took the hint which Nature gave on the first attack, and gave him an emetic, containing Zinc. sulph. ʒſs. This operated with its usual quickness, but did not seem to clear the stomach. After waiting a few minutes, I gave him the purgative which he should have taken in the morning; this very soon occasioned the complete evacuation of the stomach upwards, and he had the same instantaneous restoration of the *right* arm, as he had before experienced of the *left* arm and leg, from the emetic effect of the brandy and water. In the course of this day he was visited by Dr. Smith, from Hertford.* Blisters were applied behind the ears, a purgative in a different form repeated, and some volatile guaiacum added to his former medicines. On the 12th, a more active purgative was given, the last not having been sufficiently so. On the 13th, the blister between the shoulders was renewed, and ordered to be kept open, and pil. hydrarg. gr. iv. to be taken night and morning, and cascarilla and volatile guaiacum 8$^{a}$. quaque horâ. The loaded state of the urine with the pink sediment, and the sallowness of complexion, denoted hepatic affection, which gradually yielded to the pil. hydrarg. I particularly insisted upon the discontinuance of *tea*, of which, in his ordinary

* When Dr. Smith saw him, though he was so much better, his pulse was so irregular and indistinct, that he had some difficulty to feel it; this state of the pulse was not much mended during the complaint, and it, consequently, assisted but little in forming our prognosis.

state state of health, if he exceeded his usual quantity at breakfast, it frequently occasioned full vomiting. On the 19th, decoct. cinchon. with tinct. guaiac. ammon. was substituted for cascarilla. On the 20th, a seton on the neck was substituted for the blister, and *placed there as a permanent remedy.* On the 22d, an opiate was prescribed for him at bed-time, to the use of which I was rather reluctantly driven, in consequence of his having suffered extreme pain, not only in the seton since its insertion, but in the extremity of the fourth finger of the right hand, extending up the arm, which had deprived him of sleep ever since the 11th. The nail was black, as if blood, from a severe blow, had been extravasated under its whole surface. This proved a very painful and troublesome thing. After a few days there was a complete separation of the nail, except at its root and sides, from the sensible part beneath, which was, in fact, mortified, and which (and not extravasation) had occasioned the blackness of the nail. This dead part, after a long time, sloughed off, but there was a morbid sensibility in the contiguous parts, which continued to torment him till every particle of the nail, root and sides, was removed. A very imperfect nail has since been produced. The opiate, to the use of which we were driven by this painful complaint, procured ease and sleep, and produced no ill effect whatever.

There was a remarkable appearance of the tongue throughout the complaint; it was never dry, but the greater part of it was covered with a thin flakey white crust, an irregular patch of which seemed to have been removed, and the whole of it looked as if the mere act of drinking would wash it off. The plan of treatment above detailed, with little variation, was pursued till late in the month of April.

He had a slight attack on the 9th of June, occasioned by great fatigue and anxiety at the launch of Capt. Gower's vessel, when he very imprudently drank several cups of tea. An emetic relieved him, and he has since gradually improved in health, finding occasionally a slight degree of numbness in the *left* side. He still keeps his seton discharging, which has long since ceased to give him any pain.

I remain, Gentlemen,

*Cheshunt, Herts,* Your obliged humble Servant,

*Nov.* 10, 1812. E. HARROLD.

P. S. Should these pages meet the eye of Dr. Cheyne, whose valuable work I intend soon to see, perhaps he may be induced to favor your readers with a few remarks upon this case, which, from so very competent a judge, will add much to its value.

